# Use of Rho kinase Inhibitors in Ophthalmology: A Review of the Literature

**Published:** 2018

**Authors:** Majid Moshirfar, Lawsen Parker, Orry C. Birdsong, Yasmyne C. Ronquillo, Daniel Hofstedt, Tirth J. Shah, Aaron T. Gomez, Phillip C Sr. Hoopes

**Affiliations:** 1 John A. Moran Eye Center, Department of Ophthalmology and Visual Sciences, School of Medicine, University of Utah, 50 North Medical Dr., Salt Lake City, UT 84132, USA; 2 Utah Lions Eye Bank, Murray, UT, USA; 3 HDR Research Center, Hoopes Vision, 11820 S. State Street Suite #200, Draper, UT 84020, USA; 4 Utah Valley University, 800 West University Pkwy, Orem, UT, USA 84058, USA; 5 Kirksville College of Osteopathic Medicine, A.T. Still University, 800 W Jefferson St, Kirksville, MO 63501, USA; 6 College of Medicine, Department of Ophthalmology, University of Arizona, Phoenix, Arizona, USA; 7 School of Medicine, University of Texas, Rio Grande Valley, Edinburg, TX, USA

**Keywords:** Rho Kinase Inhibitors, ROCK, Glaucoma, Intraocular Pressure, Corneal Endothelium, Diabetic Retinopathy

## Abstract

The use of Rho Kinase (ROCK) inhibitors as therapeutic agents in ophthalmology has been a topic of discussion for several years, particularly in the realm of glaucoma, Fuchs’ endothelial dystrophy, and diabetic retinopathy. In this review, the authors provide a detailed and comprehensive overview of the published literature on the use of Rho kinase inhibitors for the aforementioned purposes. A thorough search of several databases was conducted to find sufficient literature on ROCK inhibitors. This research found strong evidence demonstrating that inhibition of Rho kinase significantly decreases IOP, increases healing of the corneal endothelium, and decreases progression of diabetic retinopathy. The main side effect of ROCK inhibitors is conjunctival hyperemia that is often present in more than half of the patients in certain formulations. Additional clinical trials investigating the reviewed treatment options of Rho kinase inhibitors are necessary to further validate previous findings on the topic. Nonetheless, it is clear that Rho kinase inhibitors have the potential to be another potent therapeutic option for several chronic diseases in ophthalmology.

## INTRODUCTION

**Function of Rho kinase**

Rho kinase is a serine/threonine protein kinase involved in the regulation and modulation of cell shape and size via action on the cytoskeleton [1]. As downstream effectors of Rho GTPase, Rho kinases are involved in calcium-independent regulation of smooth muscle contraction [2]. Furthermore, they have been linked with the control of cytoskeletal dynamics, actomyosin contractile forces, cell adhesion, cell stiffening, extracellular matrix reorganization, and cell morphology [3]. These factors have been shown to be determinants of Aqueous Humor (AH) outflow via the trabecular pathway, which consists of Schlemm’s canal, trabecular meshwork, and juxtacanalicular tissue [4, 5]. Therefore, through physiological evidence, a direct relationship is suggested between Rho kinase functionality and AH outflow passing through the trabecular pathway.

**History of Rho kinase Inhibitors**

As knowledge has been obtained regarding Rho kinases, the relationship between this enzyme and certain physiological problems has come to light. Research on Rho kinase began in the late 1990’s and has continued to the present time [1, 4, 6, 7]. The majority of research has emphasized on Intraocular Pressure (IOP) lowering the effect of Rho Kinase (ROCK) inhibitors. Fewer studies have dealt with the restorative effect a Rho kinase inhibitor has on diabetic retinopathy and the healing effects on the corneal endothelium. At present, further research is being conducted on different treatment options, dosages, and formulas for Rho kinase inhibitors in ophthalmology. In 1998, Alan Hall elucidated the relationship between the Rho pathway and actin cytoskeleton functions. He showed that the Rho kinase pathway was an important regulator of the actin cytoskeleton and that various reactions within the pathway, coordinated with many cellular responses and changed different characteristics, such as shape and adhesion.^1^ In 2001, studies began at both the University of Tokyo in Japan and Duke University in North Carolina to investigate the effects of Rho kinase inhibitors on lowering of IOP [8, 9]. They were designed to discover how AH outflow facility was increased by the ROCK inhibitors. The studies showed that, by inhibiting the Rho pathway, cells in the trabecular meshwork would alter in ways that allowed for increased outflow of AH. In the late 2000’s, studies commenced to determine if ROCK inhibitors could be used as treatment for glaucoma. Many of these studies were pioneered by the same people, who had investigated the IOP-lowering effects of Rho kinase inhibitors, namely Rao, Epstein, Vasantha, Honjo, and Tanihara, along with other collaborators [2, 9, 10].

After this period of discovery, others began research on the use of Rho kinase inhibitors as treatments for other ophthalmologic diseases. From 2010 to the present time, studies have been done to investigate the further use of Rho kinase inhibitors for different conditions, such as diabetic retinopathy and corneal endothelial damage [11]. As knowledge was gained from these investigations, further clinical trials have been performed to determine the correct formula, dosage, and duration of use of Rho kinase inhibitors [11-15]. In 2014, ripasudil, a ROCK inhibitor, gained approval in Japan to be specifically used for treatment of ocular hypertension and glaucoma [5, 16-18]. As recently as December 18th, 2017, Rhopressa, a Rho kinase inhibiting drug consisting of Netarsudil, gained Food and Drug Administration (FDA) approval; the first of its kind to do so in the United States of America [19].

**Rho kinase Signaling Pathway**

Rho kinase is a downstream effector of the RhoA protein, a small GTPase. GTPases alternate between two conformations: a Guanosine Triphosphate (GTP)-bound active conformation and a Guanosine Diphosphate (GDP)-bound inactive conformation. This GTPase activation regulation is controlled by Guanine nucleotide Exchange Factors (GEFs), GTPase Activating Proteins (GAPs), and Guanine nucleotide Dissociation Inhibitors (GDIs) [2, 3, 5, 7, 20βαFigure 1, the myosin light chain, and the LIM kinase [3].

These substrates then interact to control actomyosin contractility, membrane permeability, cellular adhesion, cell stiffening, cell morphological changes, extracellular matrix organization, as well as DNA synthesis [1, 2, 10, 21]. As mentioned previously, these cellular characteristics take on a critical part in AH outflow. Therefore, the Rho kinase has a direct role in affecting AH outflow.

**Application of Rho Kinase Inhibitors in Ophthalmology**

***Glaucoma***

Glaucoma is classified as a progressive form of optic neuropathy. There are two principal forms of glaucoma, including open angle and closed angle [4, 19]. The most predominant risk factor with either type is elevated IOP. In open angle glaucoma, it is proposed that this elevation in IOP is due to the clogging of AH drainage canal, through the Trabecular Meshwork (TM) [5]. Although the physiological mechanism for this impairment is not entirely known, it is proposed that the best therapeutic remedy for open angle glaucoma is lowering the IOP by enhancing the outflow of AH, as shown in Figure 2. Rho kinase inhibitors have been tested and proven to alter cell shape in the trabecular meshwork, allowing for enhanced AH outflow and the lowering of IOP [8, 10, 22, 23].

**Figure 1:**
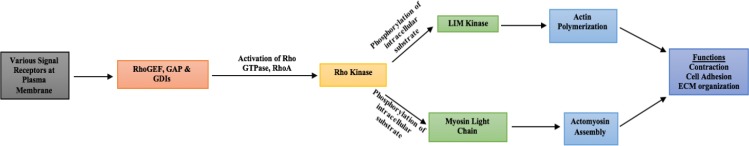
A Simplified View of Rho Kinase Involved Metabolic Pathway.

RhoGEF: Rho Guanine nucleotide Exchange Factor; GAP: GTPase Activating Proteins; GDI: Guanine nucleotide Disassociation Inhibitor; RhoA: Ras Homolog Gene Family Member A; ECM: Extracellular Matrix; GTPase: Guanosine triphosphatase

**Figure 2:**
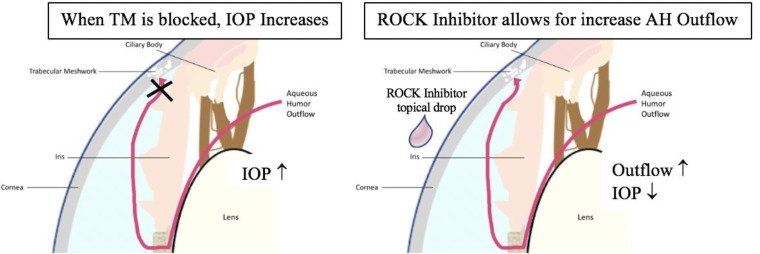
Simplified View of the Treatment of Glaucoma using ROCK Inhibitor Drops

TM: Trabecular Meshwork; IOP: Intraocular Pressure; ROCK inhibitor: Rho kinase inhibitor; AH: Aqueous Humor.

***Corneal Endothelium***

The most internal layer of the cornea is the corneal endothelium, which controls corneal hydration. It is formed by a single layer of specialized, flattened cells. Sloughing off and apoptosis of these specialized cells in Fuchs’ endothelial corneal dystrophy is one of the major causes of corneal transplantation, with other causes being ocular surgery, inflammation, and trauma [24]. Due to the wide range of cellular responses controlled by Rho kinase signaling pathway, it is hypothesized that ROCK inhibitors could play a part in both increasing cell adhesion and proliferation in the corneal endothelium [25]. This would allow for the preservation of corneal endothelial cells and the slowing of apoptosis. For this reason, the use of Rho kinase inhibitors may also help with acute corneal endothelial damage, that can potentially occur in cataract surgery [21]. Successful clinical trials have been performed to show the positive effects of Rho kinase inhibitors on the corneal endothelium [16, 21, 24].

***Diabetic Retinopathy and Macular Edema***

Diabetic retinopathy is a generalized term for disorders of the retina caused by diabetes. In the early stages of non-proliferative retinopathy, hemorrhages and vascular abnormality occur via microaneurysms and hyperpermeability of capillaries [6]. Macular edema is a progressed retinopathy, in which capillary segment walls lose the ability to control their own permeability, allowing fluid to leak in near the macula, and resulting in vision loss [26]. The proposed pathogenesis of diabetic retinopathy is related to increased leukocyte adhesion, leading to endothelial damage [27]. It has been shown that ROCK pathways promote leukocyte adhesion to microvascular structures, through increased levels of activated Intercellular Adhesion Molecule-1 (ICAM-1) and expression of other downstream proteins [28, 29]. Therefore, increased levels of activity of the Rho pathway are related to the pathogenesis of diabetic macular edema and diabetic retinopathy [27]. Treatment with ROCK inhibitor intravitreal injections would be able to reduce adhesion of leukocytes to microvascular structures, allowing for a decrease in effects of diabetic retinopathy and macular edema.

## Methods

A literature review was performed using various electronic databases, including PubMed, Science Direct, and Journal of Ophthalmology. The dates used to define the search were between January 2010 and August, 2018. For the PubMed search, Medical Subject Headings (MeSH) were used. The principal term used to dictate the MeSH search was “Rho kinase inhibitor”. It was connected to the following terms using the “AND” function: “open angle glaucoma”, “intraocular pressure”, “diabetic retinopathy”, “corneal endothelium”, “history”, and various others. The abstracts for each article were studied to ensure relevance and significance to the review. Multiple clinical studies were identified and reviewed for relevance. Sources from these studies were identified, reviewed, and included as needed. Additional searches were made to find relevant literature through MeSH, using pertinent terms on the topic. All articles deemed relevant were included in this review.

**REVIEW OF THE LITERATURE**

**Effects as an IOP-lowering Agent**

The use of ROCK inhibitors as IOP-lowering agents has been well-tested over the recent years. Many studies have been performed to determine the optimal formula and dosage. This review focused on the use of ripasudil, K-115, and netarsudil, AR-13503, due to recent approval of these two drugs for ophthalmological use in therapy of glaucoma in Japan and the United States, respectively [18, 30].

***Formula***

***Ripasudil***

Ripasudil, also known as K-115 from various clinical trials, is an ophthalmic solution used as a treatment of glaucoma. It has the chemical formula of C_15_H_18_FN_3_O_2_S and has the International Union of Pure and Applied Chemistry (IUPAC), name being 4-fluoro-5-(((2S)-2-methyl-1,4-diazepan-1-yl)sulfonyl)isoquinoline [31].

This new drug was shown to lower IOP within two hours of instillation of the drop solution, and was proven to do so consistently over a period of a full year [3, 32, 33]. However, this formula has also caused conjunctival hyperemia in the majority of subjects in each clinical trial reviewed.^26^ This ROCK-inhibiting drug gained approval in Japan, during year 2014 [10].

***Side Effects of Ripasudil***

The most commonly seen adverse effect of Ripasudil is conjunctival hyperemia. This is a dose-dependent side effect and is seen in the majority of patients treated with Ripasudil. Conjunctival hemorrhage was also seen in treated patients, however, this side effect showed no dose dependency [32].

***Netarsudil***

Netarsudil, known as AR-13503 in clinical trials, is a ROCK inhibitor with norepinephrine transport inhibitory activity, which helps lower the production of AH. It has the chemical formula of C_28_H_27_N_3_O_3_ and has the IUPAC name of (4-((1S)-1-(Aminomethyl)-2-(isoquinolin-6-ylamino)-2-oxoethyl)pheny)methyl2,4-dimethylbenzoate [34].

Netarsudil has been shown to decrease IOP within two hours of instillation and also to sustain this decrease for a 24-hour period after dosing [19]. Netarsudil uses two mechanisms to lower IOP: By acting as both a ROCK inhibitor and a norepinephrine transport inhibitor. The latter helps to prolong reduction in IOP by constriction of vascular structures in the eye. This reduces blood flow to the ciliary processes, inhibiting production of AH [30]. Netarsudil is the main component of the drug Rhopressa, which gained US-FDA approval in December 2017 [35].

***Side Effects of Netarsudil***

The most commonly seen side effect of using a netarsudil topical solution is conjunctival hyperemia. This was seen in more than half of the patients in clinical trial settings. A much smaller portion (approximately 20%) reported corneal verticillata, instillation site pain, and conjunctival hemorrhages.

***Mechanism***

Rho kinase inhibitors help lower IOP by increasing AH outflow, reducing AH production, and decreasing episcleral venous pressure (EVP) [36]. This is done in two different ways, which involves Rho kinase pathway inhibition and, as with Netarsudil, norepinephrine transport inhibition. The relationships of these variables with IOP are shown in the modified Goldman equation, which is IOP is equal to EVP added to one divided by facility of outflow (C), multiplied by the difference of formation rate of AH (F) and resorption rate of AH (U). In an equation form, this is: IOP = EVP + 1/C (F-U) [37].

***Rho Kinase Pathway Inhibition***

As previously mentioned, the metabolic pathway of Rho kinase controls many aspects of cell morphology. When inhibited, a change of cell shape and actin cytoskeleton structure occurs. After blocking the Rho kinase pathway, it has been observed that cell bodies become rounded and there is disruption of actin production [7, 8]. These two changes allow for greater outflow of AH through the trabecular meshwork, which ultimately results in a decrease of IOP.

***Norepinephrine Transport Inhibition***

Many ROCK-inhibiting drugs chemically include a norepinephrine transport inhibitor. This norepinephrine transport inhibitor helps reduce AH production and decreases EVP, which according to the modified Goldman equation, have a direct relationship with IOP [37, 38]. Norepinephrine transport inhibition lowers AH production by vasoconstriction, reducing blood flow to ciliary processes. A study showed that AH production may be reduced by 20% to 23% by the norepinephrine transport inhibitor [38]. This inhibitor affects the EVP via vasoconstriction, similar to the way brimonidine, a well-known vasoconstrictor, has been shown to lower EVP in animals [39]. The reduction in EVP accounts for more than a third of the reduction in IOP, as shown in a study using Dutch Belted rabbits [40].

***Dosage***

***Ripasudil***

A phase II clinical trial was conducted during year 2013, in Japan, that determined the optimal dosage of ripasudil, K-115. A group of individuals with open angle glaucoma were assigned to four different groups: a placebo, 0.1% ripasudil, 0.2% ripasudil, and 0.4% ripasudil.

The results of this study showed that over an eight-week period, there was a decrease in baseline IOP in all groups using ripasudil. It also showed that as concentration of dosage increased, IOP decreased. After comparing the results of the trial, researchers concluded that the optimal dosage, based on dose-response alone, was the 0.4% dose, which had a reduction in baseline IOP of -4.5 mm Hg, two hours after the last instillation of the drop. However, this study also showed that there may be a direct correlation between increased dosage and increased cases of conjunctival hyperemia. The reported cases of conjunctival hyperemia were 13.0%, 43.4%, 57.4%, and 65.3% in the placebo, 0.1% ripasudil, 0.2% ripasudil, and 0.4% ripasudil groups, respectively [32]. In Japan, a ripasudil drop solution was approved at a 0.4% concentration, as a twice daily treatment, to be used to treat glaucoma [18].

***Netarsudil***

Netarsudil, the active compound in Rhopressa, has been tested in various clinical trials to prove a dose-dependent IOP-lowering effect. In a study performed on Dutch Belted rabbits, the dose amounts of 0.005%, 0.01%, 0.02%, and 0.04% were tested, while the same study tested 0.01%, 0.02%, and 0.04% on Formosan Rock monkeys. Day three measurements of the current study had the greatest reduction from baseline IOP. The 0.04-% dose yielded the greatest lowering of IOP in rabbits and monkeys, showing a reduction of IOP of -8.1±0.7 mmHg and -7.5±1.1 mmHg, respectively. All solutions produced trace amounts of mild hyperemia in the rabbits and monkeys, yet this was the only adverse effect noted. The IOP-lowering effect lasted longer in the monkeys than in the rabbits [30].

Another study using ten humans as subjects showed the results of using a 0.02% solution over an eight-day period. Each subject received a drop of 0.02% Netarsudil solution, once a day, in one eye and a drop of the placebo in the other eye. The results of this study showed that this 0.02% solution lowered IOP from baseline by -4.6±1.8 mmHg during this eight-day period. There was a greater reduction of 3.5 to 3.6 mmHg in the netarsudil eyes than in the placebo eyes. Each subject reported mild (and in one case moderate) conjunctival hyperemia [37].

The Rho Kinase Elevated IOP Treatment trials 1 and 2 (ROCKET-1 and ROCKET-2), two phase three clinical trials, investigated safety and effectiveness relating to netarsudil and timolol in a sample of patients with elevated IOP. In a double-masked, randomized non-inferiority clinical trial, Netarsudil once a day (q.d.), produced significant lowering from baseline IOP, which was non-inferior to timolol (ROCKET-1). Netarsudil twice a day (b.i.d.) showed non-inferiority to timolol as well (ROCKET-2) [41]. In the United States, a netarsudil drop was approved in a 0.02% concentration, as a one drop q.d. treatment to lower IOP for treatment of glaucoma [35].

**Effects on the Healing of Corneal Endothelium**

Rho kinase inhibitors have been tested for use on the corneal endothelium. They allowed for increased proliferation and decreased rate of apoptosis [16]. In general, the corneal endothelium has a very limited proliferation capacity. Therefore, any structural damage is repaired by the migration of remaining corneal endothelium cells to the afflicted area, with a resulting drop in endothelium density [21]. There have been two proposed methods of delivery of ROCK inhibitors to heal the corneal endothelium, including topical eye drops and an anterior chamber injection with cultured endothelial cells [42].

***Mechanism***

**Figure 3:**
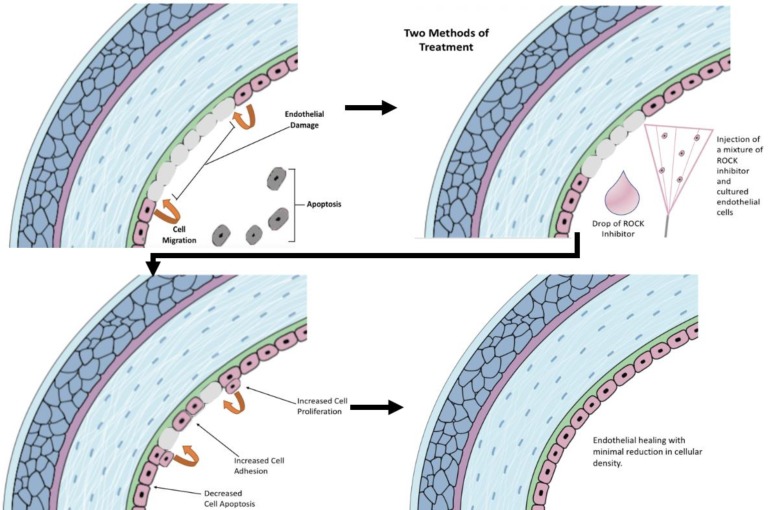
**A simplified View of the Hypothesized Treatment of Corneal Endothelial Damage using Anterior Chamber Injections and/or Topical Eye Drops of Rho Kinase Inhibitors.**

**Cell Division**

Cells in the corneal endothelium are frozen in the cell cycle. They cannot divide due to inhibiting factors in their surroundings and the tightly packed pattern of the corneal endothelium. Furthermore, ROCK inhibitors, when used to treat corneal endothelial cells, allow for increased cyclin D levels and suppression of the phosphorylation of cyclin-dependent kinase inhibitor 1B, p27/kip1, which are regulators of cell division in corneal endothelial cells. This allows for an increased rate of proliferation, as seen in Figure 3 above [21, 43].

**Slowing of Cellular Apoptosis**

ROCK is directly related to apoptosis due to the cellular responses associated with its pathway. The actin cytoskeleton contractile force, regulated by the Rho kinase, allows for cellular contraction, membrane blebbing, and nuclear disintegration. Apoptosis in the corneal endothelium can be inhibited using ROCK inhibitors, which stop this contractile force from killing the cells. It has been shown that apoptosis can be slowed within a day of using Rho kinase inhibitors [44]. This mechanism is indicated in Figure 3.

**Increased Cell Adhesion**

Cellular adhesion is a key component in the healing of the corneal endothelium. One method of healing the corneal endothelium is an anterior chamber injection of cultured endothelial cells and a Rho kinase inhibitor. Cellular adhesion allows for successful propagation of these cells to the interior layer of the cornea, as seen in Figure 3. Furthermore, ROCK inhibitors allow for increased cellular adhesion due to the enhancement of acto-myosin contractility [43]. Therefore, there is a greater potential for healing of corneal endothelial trauma using injection of cultured endothelial cells and Rho kinase inhibitors.

***Fuchs’ Corneal Dystrophy***

Fuchs’ endothelial corneal dystrophy is a progressive disease resulting in corneal endothelial cell loss. Cell death is one of the major contributing factors to the progression of this disease, affecting all layers of the cornea [42]. The current mainstay method of treatment for Fuchs is corneal transplant, however, recent studies have investigated various alternative treatment options, including the use of ROCK inhibitors.

The first treatment option is the use of a drop solution to help with the early stages of Fuchs’ corneal dystrophy and has been shown to slow cell apoptosis and increase cell proliferation [21]. These drops may also help in the treatment of late-onset Fuchs’ corneal dystrophy. One successful case was reported during year 2013 in Japan, where a male patient with late-onset Fuchs’ corneal dystrophy was treated with a ROCK inhibitor for one week. Two weeks after the treatment, corneal transparency had returned and vision had improved from 20/63 to 20/20. Another treatment option is the use of Rho kinase inhibitors as an injection with cultured corneal endothelial cells. This procedure consists of injecting a combination of cultured corneal endothelial cells and a ROCK inhibitor in the anterior chamber of the eye and then allowing the patient to lie face-down to allow the cells to be directed towards the corneal endothelium. The ROCK inhibitor facilitates increased adhesion of the cultured cells to the substrate, leading to an increase in corneal endothelial regeneration and restoration of corneal transparency [21]. This technique has been tested on rabbits and cynomolgus monkeys. The procedure enhanced the acceptance of these cells without any sign of harmful effects, such as secondary glaucoma, rejection, or problematic ectopic cell displacement [45].

***Acute Corneal Trauma***

Acute corneal trauma, which occurs during cataract surgeries, can lead to corneal degeneration. The risk for corneal degeneration increases with decreasing density of corneal endothelial cells [21]. ROCK inhibitors can be used to increase the proliferation rates of these cells in order to allow for greater density in this layer. This allows for increased healing and migration of corneal endothelial cells to cover the afflicted area.

In Japan, three patients, who had undergone cataract surgery, developed severe corneal edema and corneal haziness, and were at high risk for subsequent decompensation. Due to these conditions, they were all treated with a Rho kinase inhibitor drop. Within one to two months, there was recovery of corneal transparency, showing an increase in corneal endothelial cell density in all three patients [21].

***Effects on Diabetic Retinopathy***

The Rho pathway is involved in the pathogenesis of diabetic retinopathy, through the promotion of leukocyte adhesion to diabetic retinal microvascular structures [46]. The adhesion of leukocytes to vascular endothelium enables the release of ﻿inflammatory cytokines, growth factors, and vascular permeability factors, which ultimately compromise the blood-retinal barrier [47]. Activation of the Rho kinase pathway also has a direct correlation with microvascular endothelial damage via inactivation of the nitric oxide synthase. Inhibition of nitric oxide levels prevents vasodilation and allows for apoptosis, which increases the leukocyte-induced damage [27].

A Rho kinase inhibitor was tested on diabetic rats and was shown to decrease retinal leukocyte adhesion and to slow the corneal endothelium damage that had been caused by prior adhesion of leukocytes.^28^ In this same experiment, nitric oxide synthase production was inhibited with L-NG-Nitroarginine methyl ester, L-NAME, a well-known inhibitor of nitric oxide synthases. When this was achieved, the positive effects of the Rho kinase inhibitor were sufficiently blocked [27, 28]. It is necessary for there to be nitric oxide production in order for the Rho kinase inhibitor effects to be suffice on microvascular endothelial cells. This shows that a Rho kinase inhibitor treatment can be beneficial for patients with symptoms of diabetic retinopathy, by reducing the adhesion of leukocytes and increasing nitric oxide levels [28].

***Mechanism***

***Prevention of Creation of Anchor Sites***

**Figure 4:**
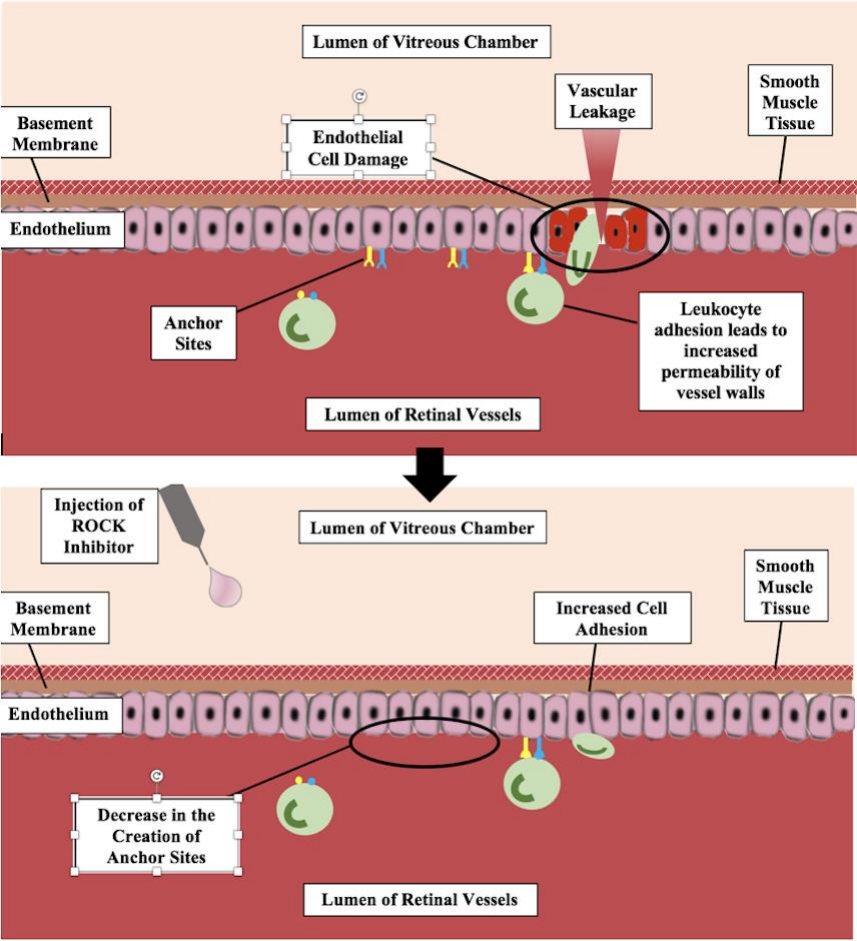
**A simplified View of the Mechanism of Rho Kinase (ROCK) Inhibitor Treatment for Diabetic Retinopathy**

Rho kinase inhibitors work to treat diabetic retinopathy by decreasing the adhesion of leukocytes and by slowing leukocyte-induced damage. Used as an intravitreal injection, the Rho kinase inhibitor slows the synthesis of various downstream proteins in the Rho pathway as well as ICAM-1. These proteins, such as ezrin and radixin, when clustered with ICAM-1, are the anchor sites for leukocytes [27, 29]. Furthermore, ROCK inhibitors stop the creation of these anchor sites, which stops further damage to the endothelium. This is indicated in Figure 4. A study from Kyushu University of Japan examined the effects of Rho kinase inhibitors related to the symptoms of diabetic retinopathy. One measured outcome was leukocyte adhesion in artificially diabetes-induced rats. After being treated with fasudil, a Rho kinase inhibitor, the rats showed a suppression of leukocyte adhesion by 68% [28]. The ROCK pathway may also contribute to damage to Müller cells induced by hypoxia and oxidative stress. Moreover, the ROCK inhibitor Y-27632 demonstrated protection of mouse retinal Müller cells against cellular injury caused by oxidative stress and hypoxia [48].

***Slowing of Microvascular Endothelial Damage***

The ROCK inhibitors also help slow down endothelial damage in the retinal microvascular vessels. They do this by reversing the endothelial nitric oxide synthase inactivation caused by the Rho pathway. This synthase creates nitric oxide, a potent vasodilator and anti-apoptotic factor [27]. Nitric oxide helps protect cells within microvascular structures without causing increased adhesion of leukocytes, which would cause further damage [27]. This permits blood flow to be restored to the cells surrounding the macula without risk of increased vessel permeability. The restoration of nitric oxide is fundamental in reducing damage caused by leukocyte adhesion to microvascular endothelial cells in the retina and for reducing apoptosis in these cells [49].

In the same investigation from Kyushu University of Japan, diabetes-induced rats, treated with Rho kinase inhibitors, showed deregulation of the nitric oxide synthase by 35% [28]. This means that the inhibition of the Rho pathway allowed for an increase of nitric oxide production. In this experiment, nitric oxide was also shown to be an integral part of the protection of endothelial cells in the retina. Furthermore, L-NAME was used to block the production of nitric oxide in these rats and, by doing so, significantly reversed the protective effect the Rho kinase inhibitor had on apoptosis in the endothelial cells [28].

***Treatment Method***

The proposed methodology of treatment for diabetic retinopathy is by an intravitreal injection of Rho kinase inhibitors. These injections will need to be frequently administered due to the short half-life of the compound in the vitreous chamber [27]. Another proposed method of administration is intravitreal implantation. This device would deliver Rho kinase inhibitors over time in order to maximize the desired effects [27]. This implantation would lessen the frequency of visits and injections for users, as proven by previous intravitreal implantations, such as Iluvien [26]. For optimal results, it is preferable to place the implantation when early symptoms of diabetic retinopathy occur, such as when microaneurysms appear. This would help slow the progression of retinal microvascular damage and maintain visual acuity [27].

***Other Uses Being Investigated***

Rock inhibitors have been investigated for the role that Rho kinase plays in the myopia scleral remodeling pathway [50].

## CONCLUSIONS

The use of Rho kinase inhibitors in ophthalmology has been studied for many different treatments. Its use has been proven to be of benefit in the treatment of glaucoma, the healing of corneal endothelium, and in progressive forms of diabetic retinopathy. Although there is evidence showing that use of Rho kinase inhibitors is related to increase in conjunctival hyperemia, the use of this new therapy can be pivotal in the treatment of ophthalmology patients around the world. More clinical trials investigating the reviewed treatment options of Rho kinase inhibitors are necessary to further validate previous findings on the topic.
